# Editorial: Control of biofilms to control caries

**DOI:** 10.3389/fcimb.2023.1332907

**Published:** 2023-11-30

**Authors:** Wen Zhou, Xiaojing Huang

**Affiliations:** Fujian Key Laboratory of Oral Diseases and Fujian Provincial Engineering Research Center of Oral Biomaterial and Stomatological Key Lab of Fujian College and University, School and Hospital of Stomatology, Fujian Medical University, Fuzhou, China

**Keywords:** caires, biofilm, antibacterial, cross-kingdom interactions, *Streptococcus mutans*

Dental caries, a disease caused by bacterial biofilms, is one of the most prevalent chronic diseases in humans globally. A dysbiotic shift at the interface of the biofilm–tooth surface leads to a prolonged low-pH environment at the interface, resulting in net mineral loss from the teeth (Zheng et al., 2023). For the prevention and treatment of caries, biofilm control is of critical importance (Comeau et al., 2022). To develop novel and effective strategies for the control of biofilms and caries, it is necessary to investigate biofilms in greater depth. Accordingly, their pathogenic mechanisms and interactions among microorganisms should be explored along with new biofilm control strategies. Herein, we discuss the issues related to biofilm control in detail.

The cariogenic mechanism of specific bacteria is an important topic in caries research. Protein lysine malonylation (Kmal) is a novel post-translational modification that is crucial for regulating metabolism and virulence expression in *Streptococcus mutans* (*S. mutans*). However, the Kmal of *S. mutans* remains poorly understood. Li et al. revealed that approximately 50% of the total malonyl lysine sites in *S. mutans* were identified as Kmal sites. It provides a multidimensional comprehension of the bacterial virulence and physiological functions of *S. mutans*. In addition, the cross-kingdom interactions of *S. mutans* and *Candida albicans* (*C. albicans*) have been found to promote the cariogenicity of *S. mutans* (Katrak et al., 2023). In this Research Topic, Li et al. reported that inhibiting the activity of glycosyl transferases (Gtfs) or glucans to hinder extracellular polysaccharide production may weaken the co-adhesion between the two species. Lu et al. summarized the coexistence and synergistic or antagonistic interactions of the two species, aiding in understanding the interactions of *S. mutans* and *C. albicans*.

There is an urgent need for developing novel and effective antibacterial agents. As part of the present Research Topic, Liu et al. revealed that areca nut essential oils reduce the cariogenicity of *S. mutans* through the targeted inhibition of Gtfs. Fang et al. explored mesoporous silica nanoparticles, which are a promising drug delivery vehicle for antibacterial agents. Furthermore, Zhao et al. reported a cold atmospheric plasma device that could easily produce plasma through a double-ring discharge structure with quartz tubes. This device was demonstrated to have a stronger bactericidal effect on caries-related biofilms than ultraviolet radiation under the same test conditions.

Achieving antibiofilm activity in a pH-sensitive way is another research hotspot. For example, Wang et al. developed a novel resin infiltrant containing a smart monomer, dodecylmethylaminoethyl methacrylate, which showed stronger antibacterial effectiveness at lower pH. Their findings shed light on the promising future of pH-sensitive anti-caries agents. In addition, Huang et al. summarized the state-of-the-art pH-activated antibiofilm strategies for the control of dental caries, focusing on their effects, mechanisms of action, and biocompatibility. The limitations of previous studies have also been discussed, thus highlighting aspects for future study.

Currently, drug-resistant strains of oral bacteria are often detected. Shen et al. reported that the fluoride-resistant strains of *S. mutans* can significantly increase the diversity of the oral microbial community. To better cope with potential drug resistance challenges in the future, there is a critical need for novel antimicrobial approaches. Based on in silico screening and computational methods, Zhang et al. used computer-aided drug design to identify compounds with optimal interactions with the target. Synthetic antimicrobial peptides obtained through rational design, computational design, or high-throughput screening have demonstrated increased selectivity for both single-species and multispecies biofilms, making the development of new antimicrobial modalities a crucial challenge for researchers in this field.

In future research, the mechanism by which *S. mutans* causes dental caries requires further exploration. For example, *C. albicans* synergistically promotes the pathogenicity of *S. mutans* through the bacterial quorum-sensing system. The use of natural products as drugs and pH-responsive antibacterial agents has great potential and is an important future Research Topic. Furthermore, there is a need to develop prevention and treatment strategies for oral cariogenic bacteria. The emergence of antibiotic resistance has necessitated the search for novel antibacterial agents that target specific oral bacterial pathogens or can be delivered through targeted drug delivery systems. Through rational design, computational design, and high-throughput screening, computer-aided drug design has led to the identification of potential therapeutic agents that can promote the targeted control of oral microbial biofilms in the near future.

## Author contributions

WZ: Formal Analysis, Writing – original draft. XH: Funding acquisition, Project administration, Supervision, Writing – review & editing.

## References

[B2] Comeau P. Panariello B. Duarte S. Manso A. (2022). Impact of curcumin loading on the physicochemical, mechanical and antimicrobial properties of a methacrylate-based experimental dental resin. Sci. Rep. 4, 12(1),18691. doi: 10.1038/s41598-022-21363-5 PMC9636433 36333357

[B3] Katrak C. Garcia B. A. Dornelas-Figueira L. M. Nguyen M. Williams R. B. Lorenz M. C.. (2023). Catalase produced by *Candida albicans* protects *Streptococcus mutans* from H_2_O_2_ stress-one more piece in the cross-kingdom synergism puzzle. mSphere 8 (5), e0029523. doi: 10.1128/msphere.00295-23 37607054 PMC10597455

[B1] Zheng T. Jing M. Gong T. Yan J. Wang X. Xu M.. (2023). Regulatory mechanisms of exopolysaccharide synthesis and biofilm formation in *Streptococcus mutans* . J. Oral. Microbiol. 15 (1), 2225257. doi: 10.1080/20002297.2023.2225257 37346997 PMC10281425

